# Analog Switching and Artificial Synaptic Behavior of Ag/SiO_*x*_:Ag/TiO_*x*_/p^++^-Si Memristor Device

**DOI:** 10.1186/s11671-020-3249-7

**Published:** 2020-01-31

**Authors:** Nasir Ilyas, Dongyang Li, Chunmei Li, Xiangdong Jiang, Yadong Jiang, Wei Li

**Affiliations:** 1 School of Optoelectronic Science and Engineering, University of Electronic Science and Technology of China, Chengdu, 610054 China; 2 State Key Laboratory of Electronic Thin Films and Integrated Devices, University of Electronic Science and Technology of China, Chengdu, 610054 China

**Keywords:** Analog switching, Synaptic characteristics, Ag/SiO_*x*_:Ag/TiO_*x*_/p^++^-Si memristor, Ag-filament

## Abstract

In this study, by inserting a buffer layer of TiO_*x*_ between the SiO_*x*_:Ag layer and the bottom electrode, we have developed a memristor device with a simple structure of Ag/SiO_*x*_:Ag/TiO_*x*_/p^++^-Si by a physical vapor deposition process, in which the filament growth and rupture can be efficiently controlled during analog switching. The synaptic characteristics of the memristor device with a wide range of resistance change for weight modulation by implementing positive or negative pulse trains have been investigated extensively. Several learning and memory functions have been achieved simultaneously, including potentiation/depression, paired-pulse-facilitation (PPF), short-term plasticity (STP), and STP-to-LTP (long-term plasticity) transition controlled by repeating pulses more than a rehearsal operation, and spike-time-dependent-plasticity (STDP) as well. Based on the analysis of logarithmic I-V characteristics, it has been found that the controlled evolution/dissolution of conductive Ag-filaments across the dielectric layers can improve the performance of the testing memristor device.

## Introduction

In 2008, Prof. Chua’s theoretical concept of memristor [1] became a reality when Strukov et al. published their studies on the relationship between magnetic flux and charge in a TiO_2_-based two compact terminal device for the first time [2], which has triggered the interests of researchers around the globe. Apart from various potential applications ranging from logic operations and reconfigurable radio frequency systems to non-volatile memory applications [2–4], memristors have also been investigated to emulate the bio-synaptic functions because of their similar structure and working dynamics. Nowadays, it is widely accepted that direct emulation of synaptic functions in an electronic device is crucial for the development of brain-inspired neuromorphic computing systems [5–7]. However, the traditionally designed electronic synapses are based on complementary metal-oxide-semiconductor (CMOS) technologies, which are suffering the von Neumann bottleneck effect in terms of the complicated execution process of computation, the limits of the integration density and energy dissipation. Therefore, the use of an adjustable two-terminal device has infused many promising opportunities to develop new structures for electronic synapses, which are resulted from the unique properties of memristors with non-volatile characteristic, nanoscale size, low power consumption, faster response, etc. [8, 9].

Recently, various materials (e.g., metal oxides like ZnO_2_, WO_*x*_, SnO_*x*_ [10, 11], chalcogenides like Cu_2_S, Ag_2_S [12, 13], and ferroelectric materials like La_2_O_3_, Pb_0.8_Ba_0.2_ZrO_3_ [14, 15]) have been investigated for the designing and fabricating of memristor devices. For many devices, change in resistance is ascribed to the field-induced migration of oxygen vacancies or metal ions (e.g., Ag^+^, Cu^2+^, and Al^3+^) and the forming of a highly conductive path. The conductive path in memristors is generally called “conductive filament (CF),” which could subsequently be broken during a switching operation. In general, two types of switching behaviors have been observed in memristors, i.e., abrupt (digital switching) and gradual (analog switching). The abrupt change in resistance is consistent with the digital signal (0 or 1), which is beneficial for the storage of information [16, 17].

In contrast to digital switching, the continuous conductance tunability in memristors bears some similarities with a biological synapse, which is the basic cellular unit for the learning and memory functions in the human brain [18]. In several memristor devices, the analog conductance changes used to emulate the weight modulation of biological synapses have been realized [19, 20]. Diffusive Ag in a-Si and oxide-based memristors with Pt/a-Si:Ag/Pt and Pt/SiO_*x*_N_*y*_:Ag/Pt structures have successfully mimicked the Ca^2+^ or Na^+^ dynamics in bio-synapses [21, 22]. Similarly, oxygen ions/vacancies filaments in metal oxide-based memristors have also been proposed for realizing synaptic functions, including short-term potentiation (STP) and paired-pulse facilitation (PPF) [23, 24]. However, biological learning rules are diverse, and up to now, not all synaptic functions have been involved in the memristor models [16].

Besides the selection of materials, the control of filament growth and rupture by inserting an additional dielectric layer in the “metal/memristive layer/metal” structure offers certain advantages to emulate synaptic functions, including STP and LTP as well as low-power consumption. Recently, in order to control the rate of conductive filament formation/rupture, Wang et al. [25] have demonstrated the analog switching behavior by inserting a SiO_2_ layer in a TaO_*x*_-based memristor. Wan et al. [26] have also realized the analog switching and emulated STP and STDP functions by inserting a reduced graphene oxide layer in the structure of Ag/SrTiO_3_/FTO memristor to control the Ag-filament overgrowth. Moreover, it has been reported [27, 28] that based on the knowledge of TiO_*x*_ material with a high dielectric constant (~40) and a low-bandgap (~3 eV), the performance of the memristor device in terms of cycle endurance and uniformity have been enhanced notably by inserting a TiO_2_ thin layer with the HfO_2_ memristive layer. Apart from this, it has been reported [24] that due to low ion mobility and low redox reaction rate, the TiO_*x*_ thin film can also act as a buffer layer to prevent the overgrowth of conductive filament, enabling a better synaptic behavior and keeping a good contact of the conductive filament during the resistive switching processes.

In this article, we report a new structure of Ag/SiO_*x*_:Ag/TiO_*x*_/p^++^-Si memristor devices and their analog switching behaviors. Compared with a single-layer device that has been reported earlier [22, 29], it has been found that the insertion of a TiO_*x*_ layer as shown in the above structure does affect the switching behavior of the memristor device in terms of enlarging conductance window and keeping a stable state during switching processes. Furthermore, the conductance of the memristor device can easily be tuned under both positive and negative pulse trains, respectively. Our recent results demonstrate that we have successfully obtained a reliable analog switching and dutifully emulated bio-synaptic functions such as short- and long-term plasticity (STP and LTP), paired-pulse facilitation (PPF) function, spike-time dependent plasticity (STDP) as well as STP to LTP transition in Ag/SiO_*x*_:Ag/TiO_*x*_/p^++^-Si memristor device.

## Methods

**Fig. 1:**
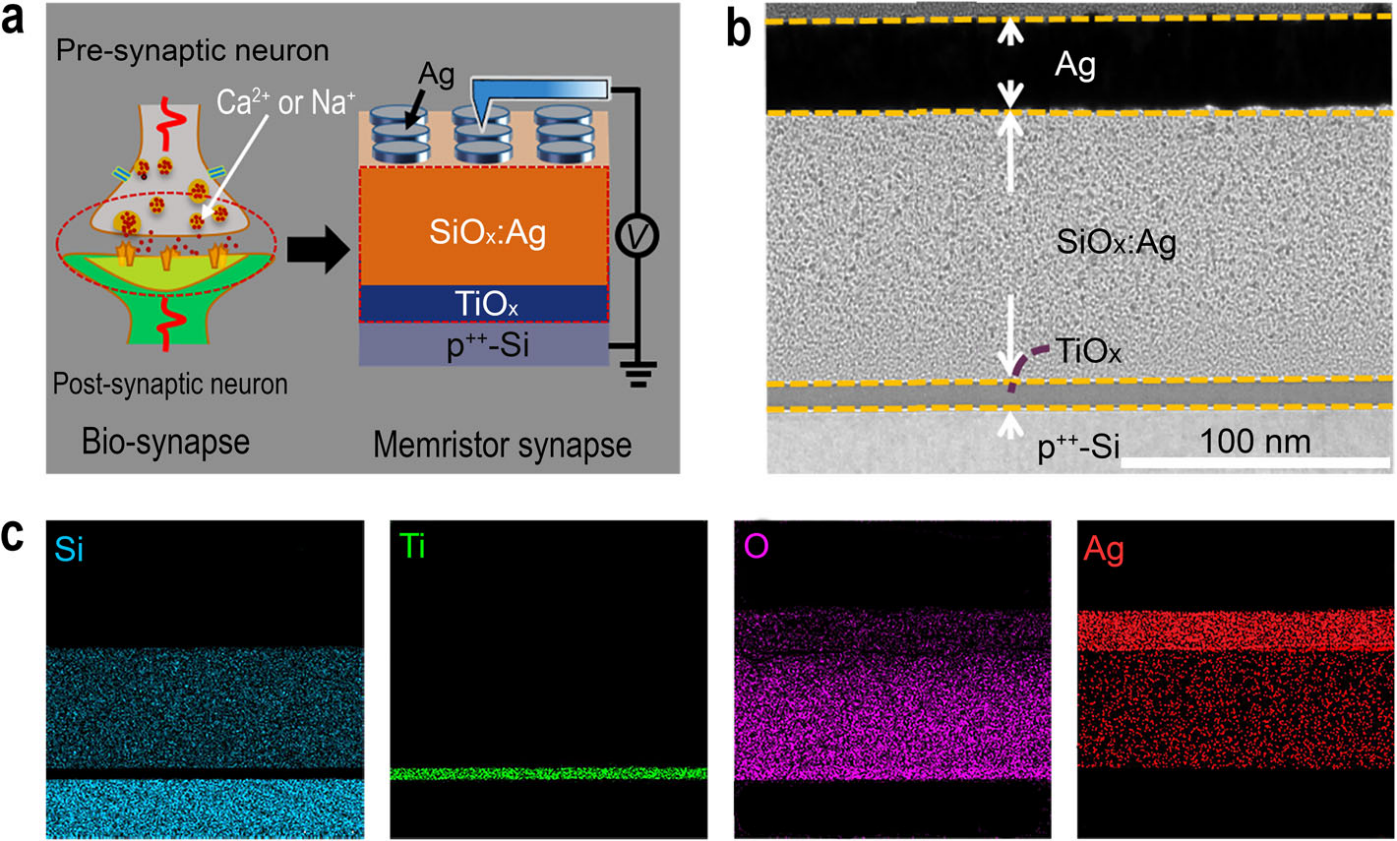
Schematic of the Ag/SiO_*x*_:Ag/TiO_*x*_/p^++^-Si memristor device and its cross-sectional TEM analysis. **a** Schematic illustration of the device and analogy between the biological synapse and electronic synapse. **b** Cross-sectional TEM view of a single memristor unit of Ag/SiO_*x*_:Ag/TiO_*x*_/p^++^-Si structure. The SiO_*x*_:Ag and TiO_*x*_ thin films sandwiched between Ag TE and p^++^-Si BE electrodes. **c** The elemental mapping of the device cross-section

i. Device fabrication: as shown in Fig. 1a, our memristor was designed as Ag/SiO_*x*_:Ag/TiO_*x*_/p^++^-Si structure. The p^++^-Si substrates (15 × 15 mm^2^) with a resistivity of about 0.01 Ω cm were cleaned by a standard method, and then the devices were fabricated on them. All the following processes were carried out at room temperature in a high vacuum system. First, a ~10-nm-thick titanium oxide layer was deposited on p^++^-Si substrates by RF magnetron sputtering using a high-purity ceramic TiO_2_ target. Then, a ~95-nm-thick SiO_*x*_:Ag layer was deposited by RF co-sputtering using a SiO_2_ target with small Ag slices placed on the magnetic sputtering path. During the deposition process, Ar flow rate and pressure were kept at 50 sccm and 20 mTorr, respectively, while the RF power was kept at 80 W. Finally, the top electrode (TE) of ~30-nm-thick Ag layer was patterned through a photolithography and lift-off technique in which the thin metal layers were deposited by using DC magnetron sputtering. The individual electrode diameter is about 150 μm.
ii. Characterization methods: transmission electron microscopy (TEM) measurements and X-ray photoelectron spectroscopy (XPS) analyses were carried out to analyze the microstructure of SiO_*x*_:Ag and TiO_*x*_ layers and the chemical state of Ag atoms, respectively, in which the TEM sample was made first by using focused ion beam (FIB, FEI Nova Nano Lab 200) and then observed under an FEI Phillips CM10- Supra TEM system. Electrical characterizations were carried out with a semiconductor analyzer (Keithley 2636B) hooked with a probe station. During the electrical measurement, the positive and negative biases were defined by the current flowing between the top electrode and the bottom one. All electrical measurements were carried out at room temperature in the air.

## Results and Discussion

A schematic of the device and the measurement configuration is described in Fig. 1a. The device has a simple structure consisting of SiO_*x*_:Ag and TiO_*x*_ thin layers sandwiched between an Ag TE and a p^++^-Si BE that is confirmed by the cross-sectional TEM of memristor cell and elemental mapping shown in Fig. 1b and c. The chemical state of Ag atoms on the surface of SiO_*x*_ is analyzed by XPS, as shown in Additional file 1: Figure S1. The Ag3d spectrum for Ag is deconvoluted to a single doublet with binding energies of 368.0 eV for Ag3d_5/2_ and 374.0 eV for Ag3d_3/2_, which are precisely assigned to Ag metallic state. The HRTEM image in Additional file 1: Figure S2 shows a cross-section of the amorphous TiO_*x*_ layer, while the small Ag nanoclusters are visible in SiO_*x*_:Ag layer, which is probably caused by the out-diffusion of Ag during the TEM sample preparation process to minimize the total interfacial energy of the material system [22]. Furthermore, the fast Fourier Transform (FFT) confirms that the Ag nanoclusters embedded in SiO_*x*_ are polycrystalline in structure, such as Ag (111) and Ag (002) nanocrystals. In the Ag/SiO_*x*_:Ag/TiO_*x*_/p^++^-Si memristor device, the Ag/SiO_*x*_:Ag and the TiO_*x*_/p^++^-Si as the pre-synaptic membrane and the post-synaptic membrane, respectively, as illustrated in Fig. 1a. The synaptic weight changes via releasing Ca^2+^ or Na^2+^ ions in a gap between pre- and post-synaptic membranes called “cleft” by the pre-synaptic membrane when the neural pulses are received. Similarly, the conductance of the Ag/SiO_*x*_:Ag/TiO_*x*_/p^++^-Si memristor device can be modulated artificially as an electronic synapse through the migration of Ag ions under the voltage impulses.

**Fig. 2:**
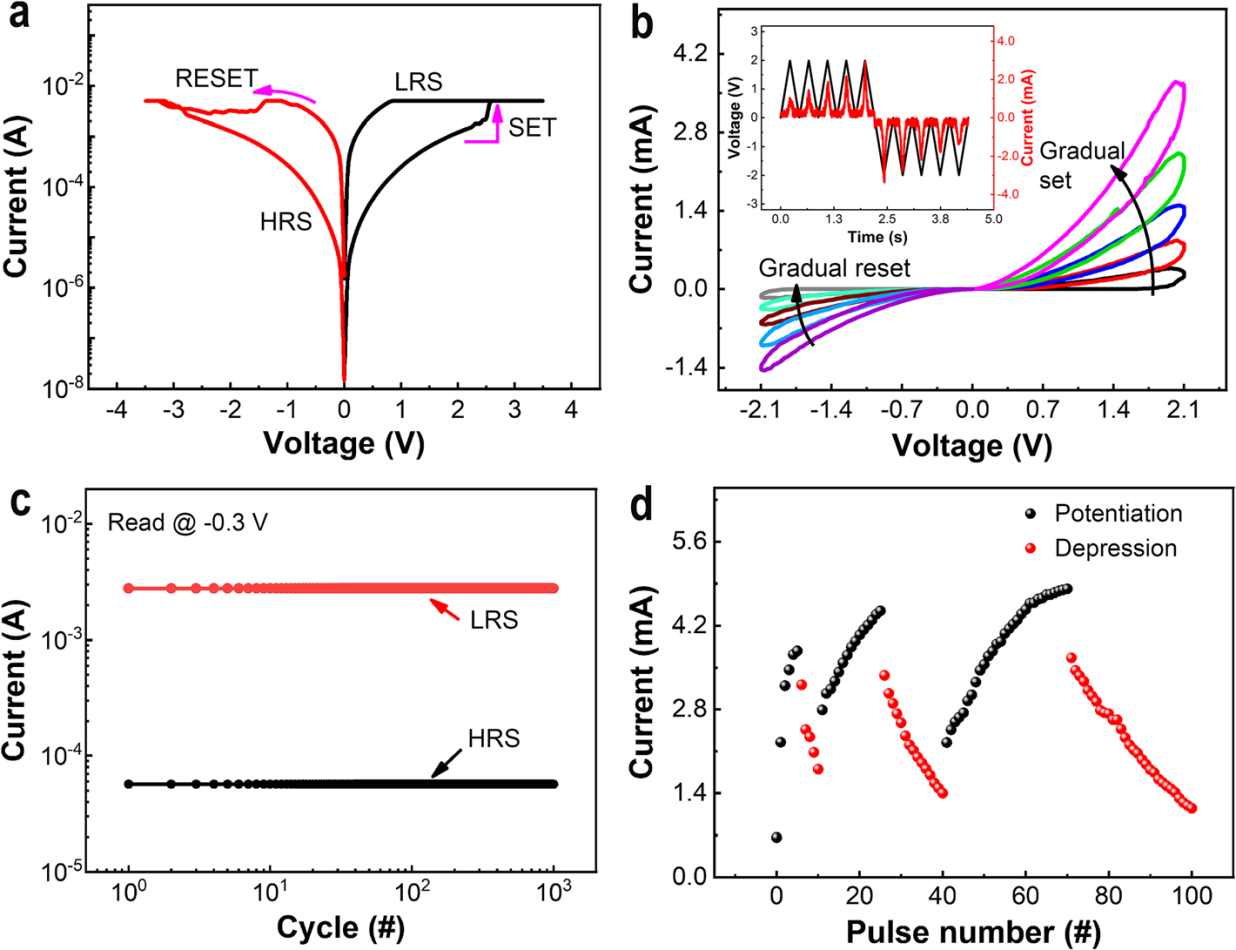
I–V characteristics of the Ag/SiO_*x*_:Ag/TiO_*x*_/p^++^-Si memristor device. **a** Bipolar switching behavior **b** Potentiation or depression by repeating voltage sweeps. The inset shows the voltage and current versus time relation (V-I-t), demonstrating the conductance state during potentiation or depression. **c** Endurance-cycling performance test at a readout voltage of -0.3 V for 10^3^ cycles of a broader range of bipolar sweeps from 0 to +4.0 V for set and 0 to −4.0 V for reset. **d** Repeated properties of conductance modulation

Figure 2a shows the current-voltage (I-V) curve of the Ag/SiO_*x*_:Ag/TiO_*x*_/p^++^-Si memristor device in the semilogarithmic scale. Under the sweeping bias of 0 V → +4.0 V → −4.0 V → 0 V, the measured I-V curve shows a pinched hysteresis loop, which is a fingerprint of a memristor. When a positive bias is applied to the Ag TE, a gradual increase in current up to the compliance current limit (I_cc_) occurs, and the resistance state of the device is changed from a high resistance state (HRS) to a low-resistance state (LRS), which is called as “SET” process. Whereas, when a negative bias is applied to the Ag TE, a decrease in current occurs, and the resistance state is returned to HRS from LRS, which is called a “RESET” process. It indicates that the device conductivity can be modulated correspondingly with a positive or negative sweep bias, showing a bipolar resistive switching behavior. Instead of an abrupt increase or decrease in current during SET and RESET processes at a high voltage regime, very interestingly, the device current consecutively increases or decreases under the repeated voltage sweep of 0 V → +2.1 V or 0 V → −2.1 V, as shown in Fig. 2b. The relation of current and voltage versus time (I-V-t) is also plotted in the inset of Fig. 2b to show the changes in conductance more clearly. As in a bio-synapse, an obvious device response of the down-up or up-down evolution of the current is observed after implementation of consecutive positive (1st-5th) and negative (6th-10th) part of I-V curves, respectively. The continuous increase (or decrease) in current during the positive (or negative) voltage sweeps indicates that the device resistance can be modulated by DC-sweeping mode. It is also observed that during each subsequent positive or negative sweep, the I–V curve picks where the last one is left off, showing a typical analog switching feature for a memristor device. The endurance performance of the memristor device can be evaluated by implementing a wider bipolar sweeping voltage at a readout voltage of +0.3 V, as in Fig. 2c, showing that the device can be operated stably and uniformly between LRS and HRS during set/reset operation over 10^3^ cycles.

The memristor device can also be operated under the pulse signals rather than DC-bias sweep voltage. Figure 2d shows the device response in the form of potentiation or depression after the implementation of repetitive potentiating (positive bias) and depressing (negative bias) pulses. The amplitudes of the potentiating and depressing pulses are +1.2 V and −1.2 V, respectively, and all the pulses widths and intervals are fixed at 5 ms. Here, the conductance modulation in the device is observed regardless of positive or negative pulse bias, which is similar to the synaptic response in the form of potentiation or depression under the potentiating and depressing stimulus, respectively. It is obviously found that the device response can be adjusted from cycle-to-cycle depending on the number of stimulation pulses, indicating that a stable and uniform potentiation and depression beyond the polarity of applied bias can be used to emulate the weight adjustment and memory enhancement in an electronic synapse [30].

**Fig. 3:**
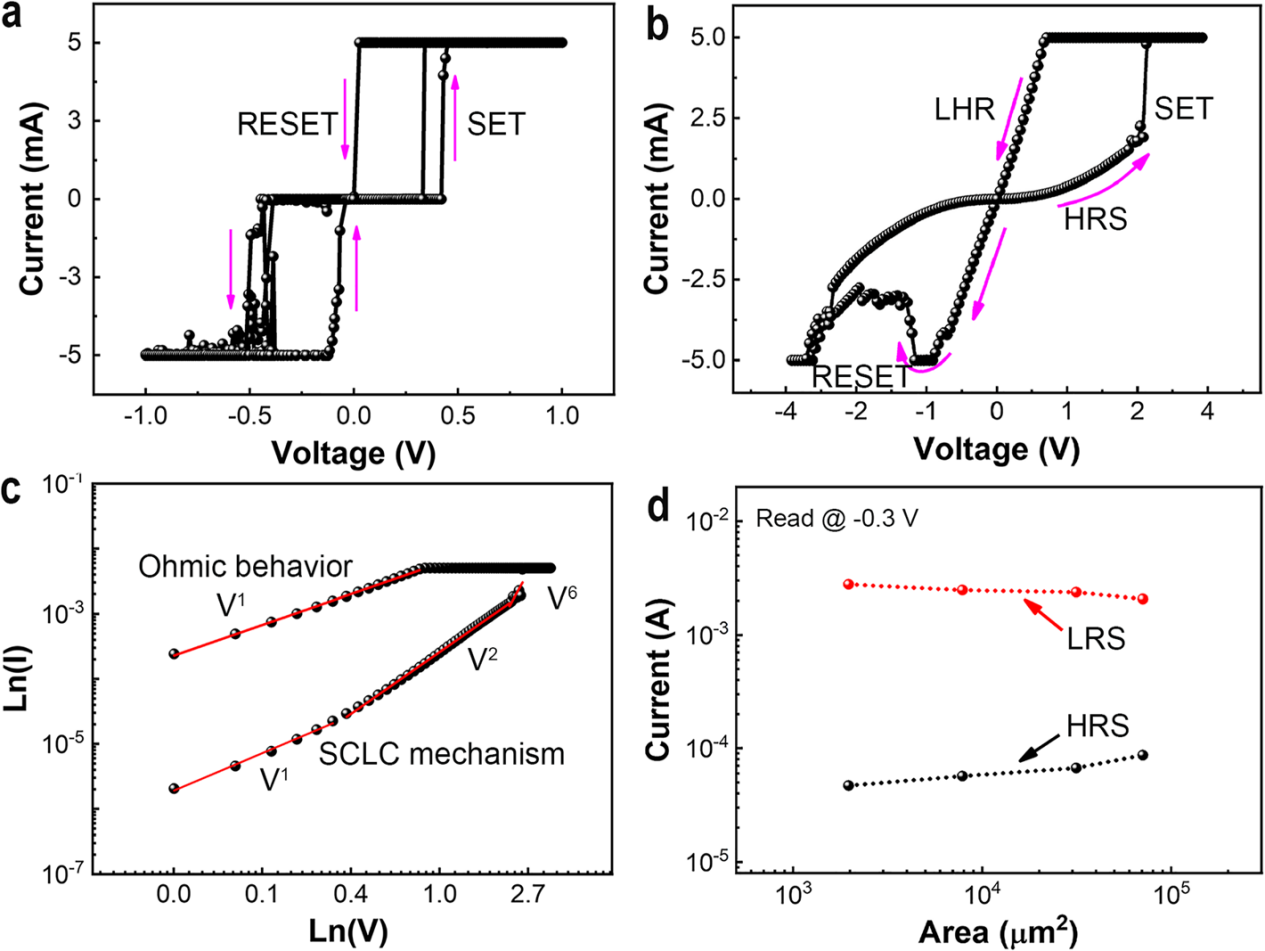
Conduction mechanism analysis of Ag/SiO_*x*_:Ag/p^++^-Si and Ag/SiO_*x*_:Ag/TiO_*x*_/p^++^-Si devices. **a** The linear I–V curve of Ag/SiO_*x*_:Ag/p^++^-Si and **b** Ag/SiO_*x*_:Ag/TiO_*x*_/p^++^-Si device. **c** The conduction mechanisms correspond to SCLC at HRS and Ohmic at LRS for the Ag/SiO_*x*_:Ag/TiO_*x*_/p^++^-Si device according to the fitting results of the positive region of I–V curve in (**b**). **d** Cell area dependence of the conductance at the LRS or the HRS

For the understanding of switching behavior, the conduction mechanisms are analyzed by fitting the I-V characteristics. For this purpose, a standalone SiO_*x*_:Ag thin-film-based memristor with the structure of Ag/SiO_*x*_:Ag/p^++^-Si is also fabricated. As shown in Fig. 3a, the device response to the quasi-DC voltage sweeps indicates a typical threshold switching behavior, as previously reported [29, 31]. The arrow directions show that the device can be cycled between the two states as volatile memory. However, the I-V curve of Ag/SiO_*x*_:Ag/TiO_*x*_/p^++^-Si memristor device shows that the situation is quite different from the standalone SiO_*x*_:Ag-based memristor device. Figure 3b shows that the device exhibits bipolar switching behaviors in aspect of the LRS and the HRS under the positive and negative part of the I-V curve, whereas the operating voltages are relatively higher. Figure 3c demonstrates the I-V curve of Ag/SiO_*x*_:Ag/TiO_*x*_/p^++^-Si memristor device, which is fitted as Ln(I) versus Ln(V) of positive region data for HRS and LRS. These fitting results show that the charge transport behavior at HRS is consistent with a classical trap-controlled space charge limited conduction (SCLC) mechanism, which consists of three portions as the Ohmic region (I/V), the Child’s law region (I/V^2^), and the steep current increase region [32]. Whereas, the linear behavior at LRS, where the slope is = 1, indicates an excellent Ohmic behavior, as shown in Fig. 3c. The different conduction behaviors at HRS and LHR are evidenced by the formation of conductive Ag-filament at LRS [32]. Figure 3d further supports that resistive switching is caused by conducting filament formation/rupture. It can be seen that while the LRS of the device is independent of the device cell size, the HRS of the device is proportional to the cell size. This size-independent property at the LRS has generally been observed in conducting filament-based memory devices [33]. Therefore, it can be concluded that the resistive switching phenomenon in Ag/SiO_*x*_:Ag/TiO_*x*_/p^++^-Si memristor device typically originates from the controlled formation/rupture of conductive filament under the positive/negative bias voltage. The gradual changes in conductance might result from the variation of the cross-sectional gap between TE and BE under the electrical field similar well to other reports [34]. So, the total resistance of the device can be described as R = Rij = V/I according to the equivalent circuit, where Rij is defined as the resistance related to the lateral gap size of CF between TE and BE. Therefore, if this gap can be adjusted through modulating the Ag CF size between TE and BE using a suitably programmed bias, then the conduction or the resistance of the memristive device can be tuned gradually.

**Fig. 4:**
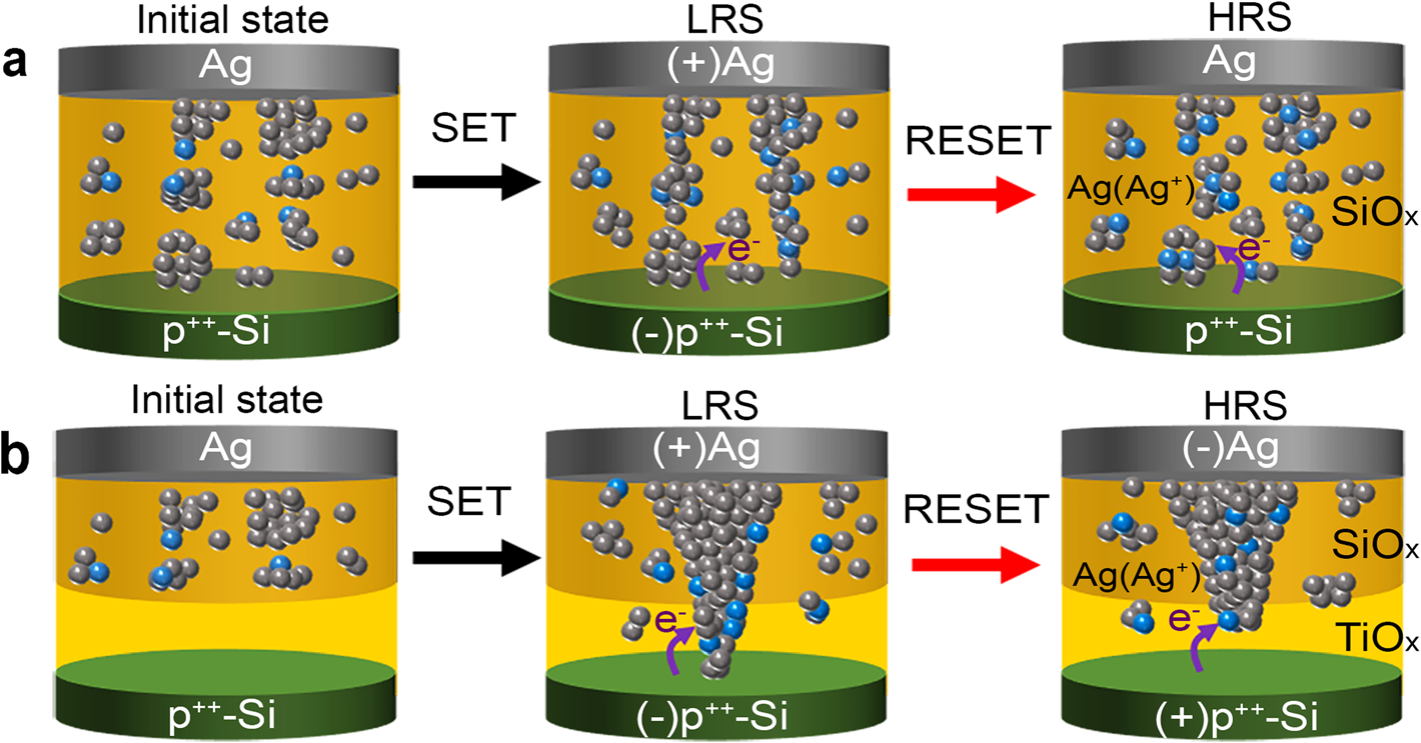
Schematic diagram of the physical model for switching behavior. **a** Ag/SiO_*x*_:Ag/p^++^-Si memristor device; **b** Ag/SiO_*x*_:Ag/TiO_*x*_/p^++^-Si memristor device

A corresponding physical model is also presented in Fig. 4 to interpret the switching mechanism in standalone SiO_*x*_:Ag and SiO_*x*_:Ag/TiO_*x*_-based memristor devices. The behavior of Ag nanoparticles in SiO_*x*_-based cells can be interpreted based on electrochemical reactions (migration and accumulation of Ag ions and Ag atoms) between the bipolar electrodes similar as reported previously [22, 35]. When the sweep voltage is applied, the Ag nanoparticles grow further to bridge the gap between the electrodes, resulting in an abrupt current increase up to the compliance level, and the memristor is turned ON in LRS (as shown in the middle panel of Fig. 4a). After removal of electrical bias, the elongated Ag nanoclusters which formed the bridge early are contracted now rapidly [22], and the device returns to HRS (as shown in the last panel of Fig. 3a) [35], indicating a bipolar threshold switching behavior in a memristor that can be cycled between the two states as in volatile memory devices.

The situation is quite different in the case of SiO_*x*_:Ag/TiO_*x*_-based memristor device, where the SiO_*x*_ thin layer has a high-bandgap (~9 eV) and a lower dielectric constant (~3), but the TiO_*x*_ layer has a low-bandgap (~3 eV) and a high-dielectric constant (~40), which makes the electric field across SiO_*x*_ layer higher than that of the TiO_*x*_ layer, dissolving more Ag atoms in the switching layer [28]. It is the low ion mobility and low redox reaction rate of titanium oxide that controls the migration and accumulation of Ag atoms and Ag ion across the interfacial layer [36]. These two facts, as mentioned above, can cause the formation of nano-cone-shaped filament from TE to BE [37]. The concentrated metallic region in the form of effective confinement of filament growth in the form of nano-cone from TE to BE can offer control of resistance states during the cyclic operation [38]. When the top Ag electrode is sufficiently positive biased across the double layers, the electric field across the dielectric layers is enough to drive the Ag ions from the Ag TE to p^++^-Si BE through the interfacial layer, leading to decrease the effective gap between electrodes (as shown in the middle panel of Fig. 4b). The Ag-filaments are not dissolved unless a negative voltage is applied and tends to maintain their original shape even the bias voltage is removed. When a negative voltage is applied, a normal RESET begins, and Ag CFs are partly desolated (usually at the thinnest location) due to the thermal-assisted electrochemical process [39]. The memristor device switches OFF and is back to HRS (last panel of Fig. 4b), and then reversibly cycled between two states (shown in Fig. 3b) as a non-volatile memory device. The left panel of Fig. 4b presents that the filaments formed here should be thicker than those in the middle panel of Fig. 4a, which cannot be dissolved and ruptured unless a negative voltage is applied. The filament part in the SiO_*x*_ layer is still much thinner than that of the nano-cone part in the TiO_*x*_ layer, and the shape of the whole filament is somehow like a nano-cone. So, when a negative bias is applied, the filament will be ruptured quickly when negative voltage is applied (Fig. 3b), whereas the voltage will be further increased and the current is again increased, indicating a risk of negative-SET at high bias range due to residual Ag atoms existing near the surface of BE.

In fact, the total memristor resistance at the HRS is just related to the gap between the filament nano-cone tip and the bottom electrode, which can be increased or decreased by adjusting the electrical bias [33]. This tendency to alter the HRS in memristors can be seen in Fig. 2b, in which the current can be increased or decreased consecutively under the repeated sweep bias from 0 V to +2.1 V and from 0 V to −2.1 V, respectively. On the other hand, the constant sweeping of a voltage under +2.1 V is not enough to form a conductive filament across the TE and BE. Instead, the conducting Ag filament can gradually accumulate Ag atoms, leading to a decrease in the effective gap between the electrodes, as shown in Additional file 1: Figure S3. Therefore, by using suitable programming bias, the transition of typical threshold switching to gradual switching can be realized, and the total resistance of the memory cell can be tuned through adjusting the effective gap between the electrodes as it can be observed in a biological synapse.

**Fig. 5:**
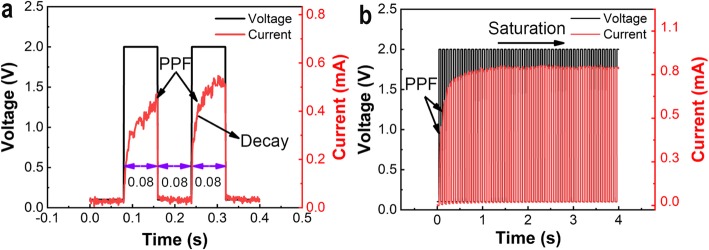
Experimental results of paired-pulse facilitation (PPF)*.* **a** Implementation of PPF on Ag/SiO_*x*_:Ag/TiO_*x*_/p^++^-Si device using +2.0 V pulse hight with 0.08 s. **b** Demonstration of synaptic weight motion of saturation using pulse train of amplitude +2.0 V with the same width and interval of 0.08 s

Similar to a bio-synapse, input stimuli with suitable pulse programming can alter the conductance states of the memristor device to perform several neural tasks. PPF is another kind of crucial feature, which can adjust conductance by temporal summation of input stimuli and perform several short-term neural tasks, including synaptic filtering and adaptation [40, 41]. PPF function in a bio-synapse works as follows: the second post-synaptic response becomes higher than that of the first one during two successive spike stimuli, leaving the interval time of spikes less than the recovery time [22]. Figure 5a shows the device response, which is monitored after implementing a pair of facilitation pulses at amplitude +2.0 V with a fixed width and interval named as a scale of 0.08 s. A noticeable increase in current as a response of the second pulse than the first one is observed, indicating an apparent change of conductance state after the implementation of suitable pulse programming. During the interval between two subsequent pulses, a current decay is observed, which can be attributed to the existence of volatile character in the device. The decay in conductance might correspond to the diffusion of Ag atoms after the removal of potentiating pulse [42]. The successful PPF function can only be executed when the time interval between two consecutive pulses is less than the diffusing relaxation time of Ag atoms, causing more Ag atoms pushed in the SiO_*x*_:Ag/TiO_*x*_ layer. Moreover, a saturation state is achieved when the device is continuously stimulated with a number of facilitation pulses with amplitude +2.0 V and a fixed width and interval named as a scale of 0.08 s, as shown in Fig. 5b. The results show that when high-frequency pulses are applied, which pumps more Ag atoms in the SiO_*x*_ layer until a conducting bridge is formed across the TE and BE, achieving a saturation level [22]. This phenomenon is quite similar to the Hebbian learning rule, where the synaptic weight changes must have a saturated value to avoid excessive excitability of neurons with the unstained spikes of pulse train applied [43].

**Fig. 6:**
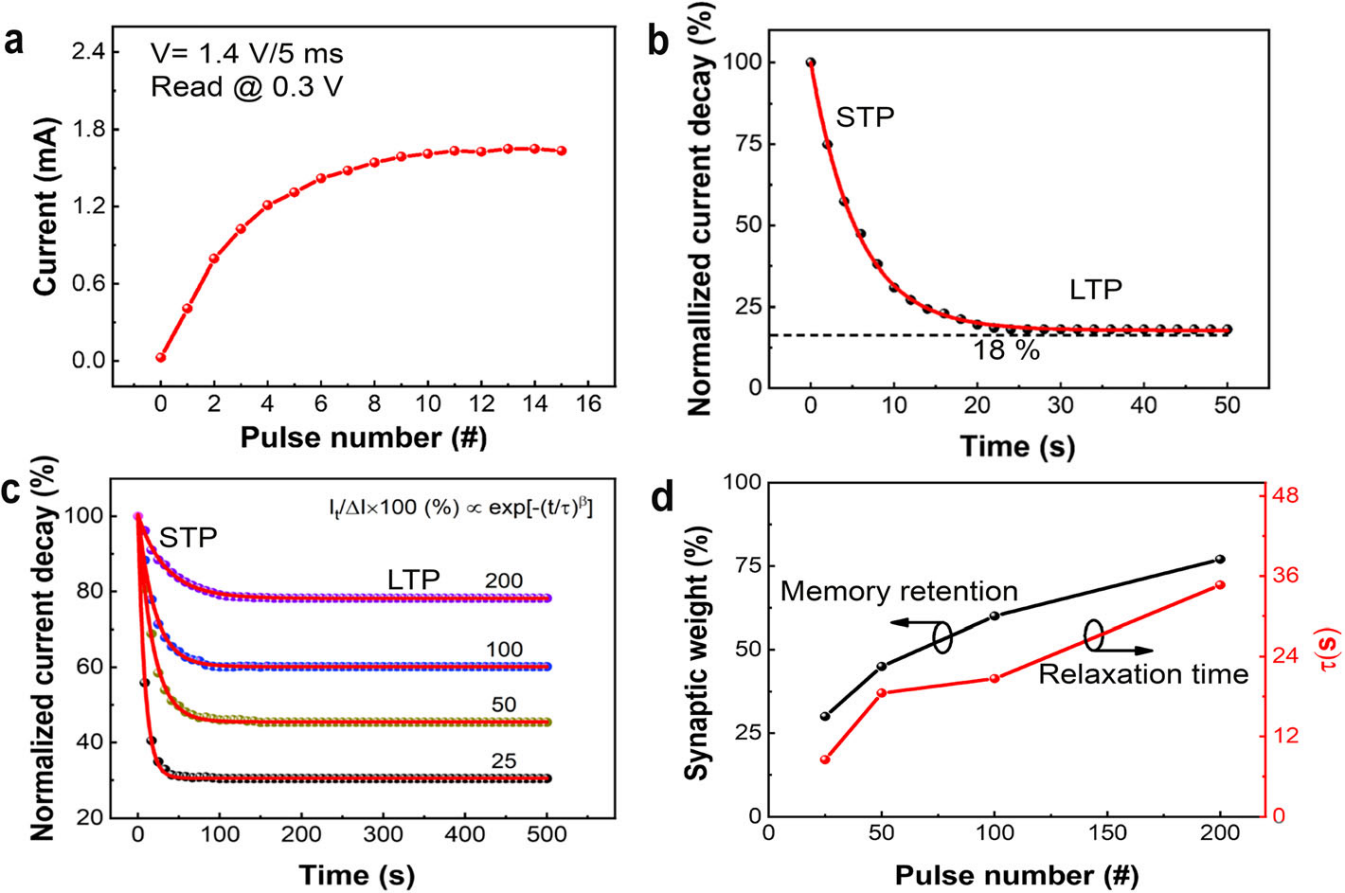
Current decay and memory retention in Ag/SiO_*x*_:Ag/TiO_*x*_/p^++^-Si memristor. **a** Increase in current after implementation of consecutive 15 potentiating identical pulses. **b** Current decay is monitored at a reading voltage of +0.3 V after stimulating the device. **c** The normalized current decay versus time showing the transition from STP to LTP after implementation pulse trains of the number of stimulation. **d** The memory retention and relaxation time (*τ*) to pulse numbers

Furthermore, the same as in a biological synapse, a memristor will suffer a memory loss with a sudden decrease in current after implementation of potentiating spike, which can be ascribed as the existence of STP in memristor [44, 45]. In neurobiology, STP and LTP are commonly ascribed as short-term memory (STM) and long-term memory (LTM) [46]. It has been established that the STP to LTP transition could occur through the repeating stimuli (i.e., a process of rehearsal) [46, 47]. In order to verify and compare this behavior with those observed in biological synapses, a sequence of voltage pulses has been implemented to our Ag/SiO_*x*_:Ag/TiO_*x*_/p^++^-Si memristor devices. Figure 6a shows the increase of current from an initial state of 0.05 mA to 1.8 mA after implementation of 15 consecutive pulses (amplitude +1.4 V, width and interval 5 ms). The normalized current decay ((I_t_-I_o_)/I_o_ × 100%) is measured at reading voltage +0.3 V immediately after imposing potentiating pulses with time (*t*), as shown in Fig. 6b. The relationship between the normalized current decay and time well fitted by the relation given in Eq. (1) [48]:

1 $$ {\Delta  I}_t/{\Delta  I}_o\times 100\%=\exp \left[-{\left(t/\tau \right)}^{\beta}\right] $$

here, *τ* is called relaxation time, and *β* is called the stretch index (0 < *β* < 1). Generally, this relation is used to describe the relaxation processes in disordered materials with a random distribution of energies. The curve shows that the decay in synaptic weight is similar to the human memory “forgetting curve” in psychology [49], which features a fast decay at the beginning that could be called STP and then gradually achieves a stable level that could be called LTP in the device. However, an obvious decay of the normalized current in the device is observed in a very short interval of time (*t*) and then achieves a saturation level at a low conductance state (up to 16% in 50 s). Under this situation, without any other change in pulse parameters, we have carried out a further simulation process with a repeated number of pulses. The current is measured at a read voltage of +0.3 V immediately after imposing different numbers of pulses (i.e., 25, 50, 100, and 200) from the same initial state for each set of pulse trains. Figure 6c shows that the normalized current decay with time in each set of measurements is fitted by the relation given in Eq. (1). Figure 6d shows that by an increase of the stimulation number, the relaxation time (*τ*) increases, indicating forgetting process fades slowly. Meanwhile, an obvious elevation of the current level is observed, implying a positive change in synaptic weight (conductance), as shown in Fig. 6d by the black line. These results presented in Fig. 6 provide clear evidence of the existence of STP and LTP in our device. A smaller number of stimulations can only induce STP in the device, with a slight rise in conductance at saturation level. Therefore, by increasing the number of repetitive stimulations, the rehearsal process not only rises a conductance level but also is achieved a long-lasting memory retention phenomenon, as shown in Fig. 6d by the red line.

The conventional digital-type memories require the non-volatility to store the information, while in bio-synapse, information processes and accordingly reconfigures the memory status. It can be seen in Fig. 6 that the transition from STP to LTP is realized, and the synaptic weight is adjusted accordingly, while the unimportant ones with small synaptic weight are temporarily stored and then diminished with time. This phenomenon is quite similar to the “multi-store model” presented by Atkinson and Shiffrin [50]. In this model, first input information is analyzed, then stored in different hierarchy levels, according to the importance of “synaptic weight” through the rehearsal process. Therefore, an increase in synaptic weight and resultant prolonged relaxation time (*τ*) in our device as a function of stimulation numbers has best verified the rehearsal scheme.

**Fig. 7:**
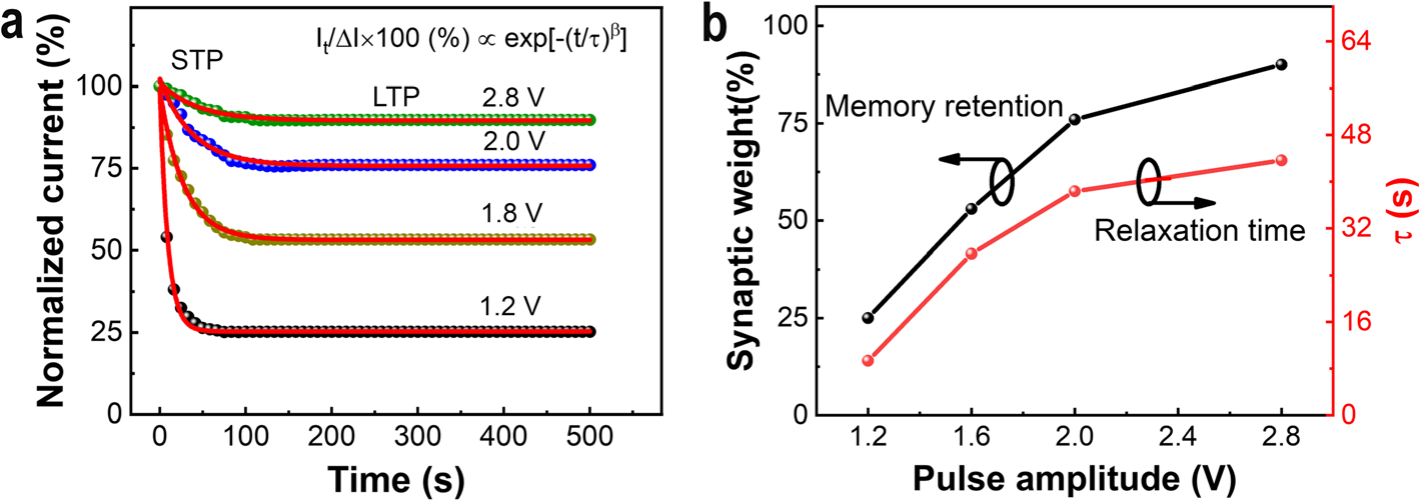
Experimental results of current decay in Ag/SiO_*x*_:Ag/TiO_*x*_/p^++^-Si memristor device after the stimulation process. **a** The normalized current decay versus time showing the transformation from STP to LTP; **b** The memory retention and relaxation time (*τ*) as a function of the pulse amplitude

Besides the pulse repetition process, the transition of the STP to the LTP phenomenon is further examined as a function of pulse strength. The device response is monitored after implementation of different pulse amplitudes, i.e., +1.2 V, +1.6 V, +2.0 V, and +2.8 V with similar width and interval scale of 3 ms, as shown in Fig. 7a. The current is monitored with a readout voltage of +0.3 V after imposing each pulse train consisting of 50 pulses. The fitted results with the stretched exponential relaxation model in Fig. 7a shows that the relaxation time is increased as a function of pulse strength (as shown in Fig. 7b red line). Meanwhile, as shown in Fig. 7b, an elevation of the synaptic weight of about 90% is observed at a larger *τ* of 43 s and +2.8 V amplitude, which is much higher than the synaptic weight of about 25% at a smaller *τ* of 10 s and +1.2 V amplitude (as shown in Fig. 7b black line), indicating the formation of LTP. Based on these results, it is easy to find that the formation and persistence of LTP in our device are highly dependent on both pulse numbers or pulse amplitude. These results coincide with the facts that the memory states, i.e., STM and LTM, and their stabilities in bio-synapses are related to the input stimulus characteristics.

**Fig. 8:**
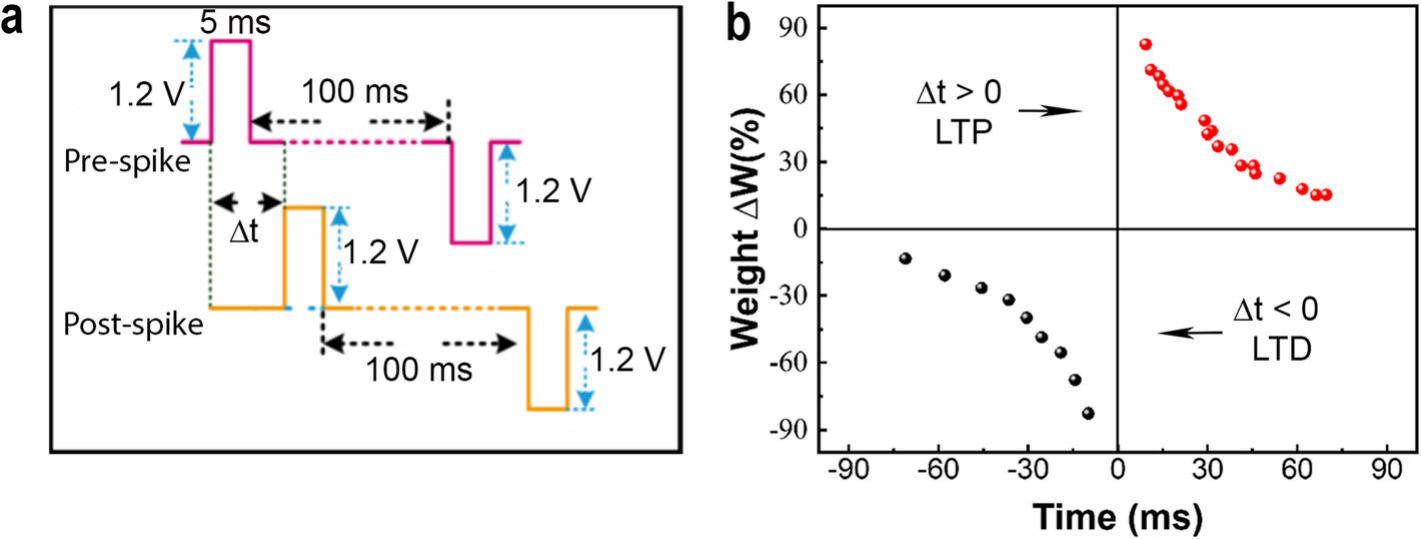
Experimental results for implementation of STDP rule in Ag/SiO_*x*_:Ag/TiO_*x*_/p^++^-Si memristor device. **a** The schematic illustration of implementing electrical programming bias comprising the pair of pulses at amplitudes +1.2 V and −1.2 V fixed with the same width of 5 ms. The approaching time difference between stimulus pulses is Δt ms (t = ±10n, *n* = 1, 2, …, 10); **b** The synaptic weight (ΔW) as a function of spike timing (Δt), demonstrating well on the potentiation and depression behaviors in the memristor device

The spike-time-dependent-plasticity (STDP) is another fundamental character for learning and memory function [51] in a biological synapse. It has been reported [52] that in the electronic synapse, the weight can be modulated by a relative timing of pre- and post-synaptic pulses. The Hebbian STDP rule works as follows: if the pre-spike precedes the post-spike (Δt > 0), it could reinforce the connection strength between two neurons. In contrast, if the post-spike heads the pre-spike (Δt < 0), it could weaken the connection strength between two neurons. Such kinds of reinforcement and weakening of connection strength between two neurons are also called LTP and LTD, respectively [45]. In the whole process, the order of pre- and post-spikes with respect to time determines the weight change (ΔW) polarity. In order to emulate the STDP rule in our device, a pair of pulses (+1.2 V, 5 ms, and −1.2 V, 5 ms) as pre- and post-spiking signals are implemented, as shown in Fig. 8a. It can be seen that there will emerge a more considerable conductance change (synaptic weight) with the decrease of Δt (in both cases when Δt > 0 and Δt < 0). The percentage change in synaptic weight is defined as ΔW = (G_t_−G_0_)/G_0_ × 100%. Here, G_0_ is the conductance measured before stimulation and G_t_ is the conductance measured after the stimulation using pre- and post-spiking pairs, respectively. A plot is shown in Fig. 8b, that can explain the relationship between ΔW and Δt before and after the simulation process. It can be seen that when the pre-synapse (positive) appears before the post-spike (negative) (Δt > 0), the conductance is enhanced with an increase in ΔW along with the decrease in Δt. On the contrary, when the pre-synapse (positive) appears after the post-spike (negative) (Δt < 0), the net conductance of the device is decreased (depressed) in ΔW along with the increase in Δt. These results have demonstrated that our Ag/SiO_*x*_:Ag/TiO_*x*_/p^++^-Si memristor device has successfully emulated the Hebbian STDP learning rule in the form of an artificial synapse.

## Conclusions

In summary, a new kind of memristor device with the simple structure of Ag/SiO_*x*_:Ag/TiO_*x*_/p^++^-Si has been fabricated by a physical vapor deposition process. The synaptic characteristics of the memristor with a wide range of resistance change for synaptic weight modulation by implementing positive or negative pulse trains have been investigated extensively. Several crucial learning and memory functions have been demonstrated simultaneously in such a single fabricated memristor device, including short-/long-term potentiation and depression (STP/STD, LTP/LTD), PPF and the STP-to-LTP transition as well as STDP, which are adjusted and controlled by repeating pulses more than a rehearsal operation. Furthermore, the analysis of logarithmic I-V characteristics with corresponding physical model indicates that the controlled formation/dissolution of Ag-filaments across the Ag and p^++^-Si electrodes could improve the performance of the new Ag/SiO_*x*_:Ag/TiO_*x*_/p^++^-Si memristor device with a buffer layer of TiO_*x*_ between the SiO_*x*_:Ag layer and the bottom electrode. This developed device, as an artificial synapse, might bring a potential research prospect in the design and hardware implementation of new-generation biomimetic neural networks and computing systems.

## Supplementary information

**Additional file 1.** Supporting information.

## Data Availability

All data are fully available without restriction.

## Abbreviations

BE: Bottom electrode
HRS: High-resistance state
I-V: Current-voltage
LRS: Low-resistance state
LTM: Long-term memory
LTP: Long-term plasticity
PPF: Paired-pulse-facilitation
SCLC: Space-charge limited conduction
STDP: Spike-time-dependent-plasticity
STM: Short-term memory
STP: Short-term plasticity
TE: Top electrode
TEM: Transmission electron microscopy
XPS: X-ray photoelectron spectroscopy
