# Supplementary material for: Analog Switching and Artificial Synaptic Behavior of Ag/SiOx:Ag/TiOx/p++-Si Memristor Device
Source: Nanoscale Res Lett. 2020 Jan 31;15:30. doi: 10.1186/s11671-020-3249-7 (PMC6994582; doi:10.1186/s11671-020-3249-7)
Supplement: Supplementary file 1 — Additional file 1. Supporting information. [file 11671_2020_3249_MOESM1_ESM.doc]

**Analog switching and artificial synaptic behavior of Ag/SiO*_x_:*Ag/TiO*_x_*/p^++^-Si memristor device**

**Nasir Ilyas^1^, Dongyang Li^1^, Chunmei Li^1^, Xiangdong Jiang^1^, Yadong Jiang^1,2^, Wei Li^1, 2*^**

^1^ School of Optoelectronic Science and Engineering, University of Electronic Science and Technology of China, Chengdu 610054, China

^2^  State Key Laboratory of Electronic Thin Films and Integrated Devices, University of Electronic Science and Technology of China, Chengdu, 610054 China.

* Correspondence: [wli@uestc.edu.cn](mailto:wli@uestc.edu.cn)

**Supporting information**


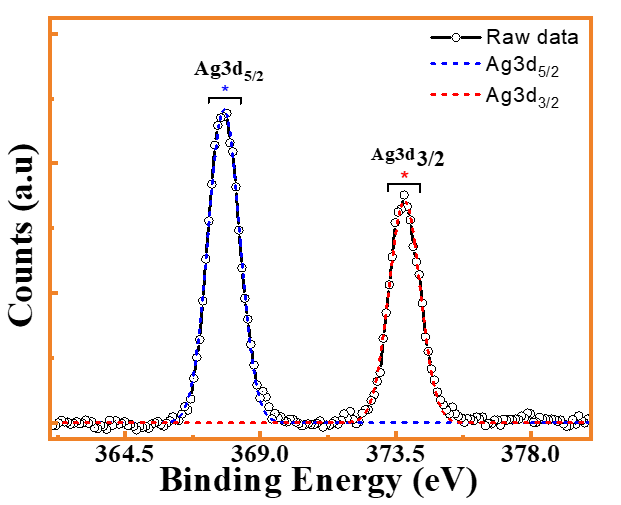


Figure S1. XPS analysis of Ag atoms at the surface of SiO_x_ layer


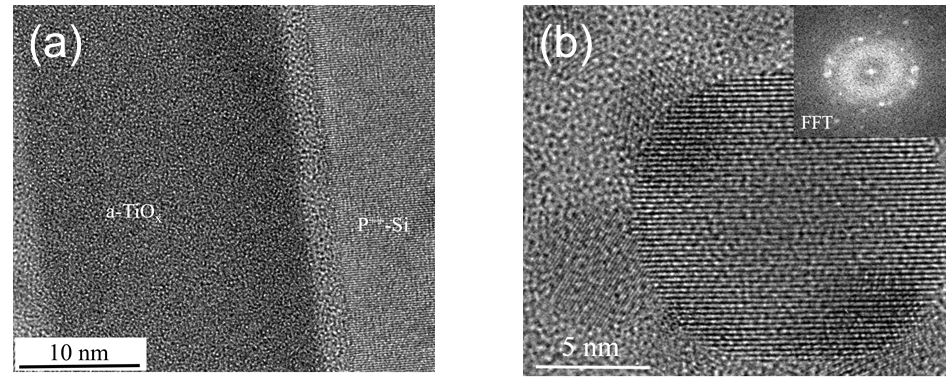


Figure S2. HRTEM image of TiO*_x_* (a) and SiO*_x_*:Ag (b) thin layers. The inset in figure shows the FFT analysis of Ag nanoclusters.


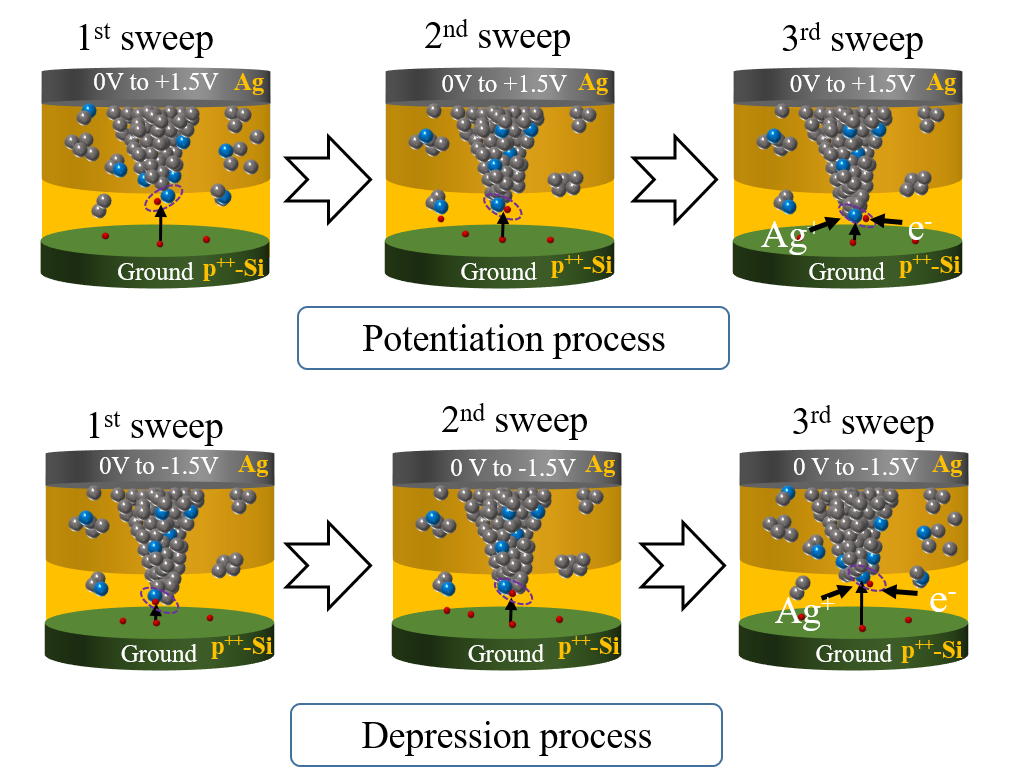


Figure S3. Illustration of potentiation (above) and depression (below) process by repeating the positive and negative voltage sweeps
